# Between-Leg Mechanical Differences as Measured by the Bulgarian Split-Squat: Exploring Asymmetries and Relationships with Sprint Acceleration

**DOI:** 10.3390/sports5030065

**Published:** 2017-09-01

**Authors:** Robert G. Lockie, Fabrice G. Risso, Adrina Lazar, Dominic V. Giuliano, Alyssa A. Stage, Tricia M. Liu, Megan D. Beiley, Jillian M. Hurley, Ibett A. Torne, John J. Stokes, Samantha A. Birmingham-Babauta, DeShaun L. Davis, Ashley J. Orjalo, Matthew R. Moreno

**Affiliations:** 1 Department of Kinesiology, California State University, Fullerton, CA, 92831, USA; fabricerisso@csu.fullerton.edu (F.G.R.); deshaunl.davis@csu.fullerton.edu (D.L.D.); ashley.orjalo@csu.fullerton.edu (A.J.O.); moreno.matthewr@csu.fullerton.edu (M.R.M.); 2 Department of Kinesiology, California State University, Northridge, CA, 91330, USA; adrina.lazar.957@my.csun.edu (A.L.); dominic.giuliano.871@my.csun.edu (D.V.G.); alyssa.stage.634@my.csun.edu (A.A.S.); tricia.tomita.228@my.csun.edu (T.M.L.); meganbeiley@gmail.com (M.D.B.); jillian.hurley.288@my.csun.edu (J.M.H.); ibett.torne.734@my.csun.edu (I.A.T.); john.stokes.91@my.csun.edu (J.J.S.); samantha.birminghambabauta.162@my.csun.edu (S.A.B.-B.)

**Keywords:** force, power, velocity, single-leg, sprinting, unilateral strength

## Abstract

Between-leg strength differences can negatively influence sprint acceleration. The challenge is to find a method to measure this within a unilateral exercise. This study analyzed a five repetition-maximum (5RM) Bulgarian split-squat (BSS) to identify between-leg differences for the dominant and non-dominant legs in peak and mean power, force, and velocity as measured by a linear position transducer. Between-leg differences in these variables were correlated with 20-m (0–5, 0–10, 0–20 m intervals) sprint velocity. Eight men were assessed in the 5RM BSS and 20-m sprint. T-tests calculated between-leg differences in power, force, and velocity. Spearman’s correlations calculated relationships between the between-leg differences in the mechanical variables with velocity over each interval. When comparing the dominant and non-dominant legs, there were significant (*p* = 0.002–0.056) differences in 11 of 12 variables. However, percentage differences were low (~0.3–12%). There was one large, non-significant correlation (best repetition mean force between-leg difference and 0–5 m velocity; *ρ* = −0.810) out of 36 relationships. The BSS can provide a profile of between-leg differences in power, force, and velocity. There were limited relationships between the BSS between-leg differences and 20-m sprint velocities. Smaller between-leg differences in BSS power, force, and velocity could ensure minimal impact on acceleration.

## 1. Introduction

Many athletes need to have the ability to rapidly accelerate, as the nature of most court and field sports do not often permit the opportunity to complete sprints over the distances required to achieve maximum speed [1,2,3]. Numerous physical capacities contribute to sprint acceleration over short distances, including: strength and force generation [2,4,5]; power and rate of force development [4,6,7]; dynamic stability [8]; and sprint acceleration technique [3,9,10,11]. Lower-body strength in particular is important for acceleration, as the individual must be able to generate enough force to overcome the inertia of their body mass. The influence that strength has on sprint acceleration can be investigated through the use of correlation analyses between sprint tests and exercises such as the back squat [3,4,5,6]. However, an exercise like the back squat is bilateral, and thus does not provide information about any strength differences between the legs. This is pertinent, as between-leg differences or asymmetries in force production can negatively impact running speed [12]. Given that sprinting is a cyclic activity alternating between unilateral support and flight [9,11], there would be value in understanding the strength characteristics of each leg, and how this may influence sprint acceleration. Coaches have a definite need to understand the strength characteristics and strategies adopted by athletes when accelerating from each leg, as this will help them define if and why an asymmetry exists [13].

There has been some analysis of between-leg strength differences in the literature, and this has typically been conducted using isokinetic dynamometry [14,15,16]. This type of analysis can potentially document how asymmetries between the legs could affect athletic performance. For example, Lockie et al. [15] investigated bilateral differences in the knee extensors and flexors in male recreational team sport athletes, and ascertained whether they influenced multidirectional speed. Interestingly, Lockie et al. [15] found that greater between-leg differences in knee extensor torque and knee flexor work measured at 240 degrees per second actually related to faster times in a 40-m sprint. However, it has been noted in previous research that between-leg strength differences of less than 15% are generally not functionally significant [17,18]. Lockie et al. [15] found the between-leg differences between knee extensor torque and knee flexor work of their participants were less than 10%. As a result, Lockie et al. [15] suggested that as long as any asymmetries are not too great, there should not be an impact on running speed. While these results are notable, there are limitations associated with the use of isokinetic dynamometers. The equipment is expensive, data collection can be time-consuming, and it is not always practical to use isokinetic strength testing on large squads of athletes. Further to this, isokinetic dynamometers tend to isolate muscle groups. The sprint step during acceleration is a multi-joint leg movement [1], and thus a unilateral strength measure that takes this into account would appear beneficial when investigating asymmetries. It would be also be more advantageous for the coach to be able to measure between-leg strength differences in a more practical environment such as the gym, and to do so in a time-efficient manner (i.e. during a training session).

Linear position transducers have become very popular in recent years for athlete monitoring during strength exercises, and have great application for the strength and conditioning coach or sport scientist [19,20,21,22]. A linear position transducer features a cord that can be attached to the bar, which measures variables such as the power and force applied to the bar, and the resulting bar velocity during lifts. In this way, the coach can be provided with information about how a load is being moved during an exercise, as opposed to just the absolute load being lifted. This would have application for a unilateral strength exercise, as a coach or scientist can measure the performance of each leg with the same load, and whether there are between-leg differences in the key mechanical characteristics. The Bulgarian split-squat (BSS), which involves an individual performing a single-leg squat under load while the non-working leg is supported on a bench [23], is an example of an exercise where this analysis could take place. Although balance may influence the performance of this exercise, in a wide-ranging literature review and meta-analysis, Muehlbauer et al. [24] noted that balance and the ability to produce force are relatively independent qualities and are task-specific. Thus, the BSS performed with a relatively heavy load will stress an individual’s strength capacity more than their balance [24]. In addition to this, because of the unilateral focus, the BSS has been recommended for speed training [25,26], so it should have application for sprint acceleration. The actions required in the BSS (flexion of the hips, knees, and ankle during the descent, and extension in the ascent) would appear to more closely match the actions required in the sprint step [1,27,28,29,30], and much more so than isokinetic strength measures. Accordingly, the performance of each leg within the BSS could provide the coach with information about any mechanical between-leg differences that could influence an athletic activity such as sprint acceleration. The use of the BSS as a potential measure of between-leg asymmetries has not been analyzed in the current literature, nor has the relationships of asymmetries measured by this exercise with an action such as sprint acceleration been investigated.

Therefore, this initial exploratory study had two main goals. The first was to provide a preliminary analysis of the five repetition-maximum (5RM) BSS to identify between-leg differences in mechanical characteristics such as peak and mean power, force, and velocity in strength-trained males. This load was selected as it has been recommended and used in lower-body strength training [31,32,33]. The second goal of this study was to investigate the relationship between lower-limb differences in mechanics as measured by the 5RM BSS with sprint acceleration over 20 m, including the 0–5 m, 0–10 m, and 0–20 m intervals. It was hypothesized that the 5RM BSS could be used to identify between-leg differences in mechanical characteristics such as power, force, and velocity. A further hypothesis was that smaller between-leg differences in power, force, and velocity as measured by the 5RM BSS would relate to faster velocities over the sprint intervals.

## 2. Materials and Methods

### 2.1. Subjects

Eight strength-trained men (age = 23.43 ± 1.51 years; height = 1.77 ± 0.05 m; body mass = 77.76 ± 10.30 kg) were recruited for this study. Participants were required to: be currently strength training (≥ three hours per week); have a strength training history (≥ two times per week) extending over the previous year; and not have any medical conditions compromising participation in the study. All participants were familiar with the BSS and maintained their normal physical activity and diet for the duration of the study. The methodology was approved by the institutional ethics committee. Participants received an explanation of the study, including the risks and benefits of participation, and written informed consent was obtained prior to testing.

### 2.2. Procedures

Two testing sessions were utilized in this study, which were completed over a two-week period. Depending on participant availabilities, 48–72 h were provided between testing sessions. The first session involved an assessment of the 5RM BSS, while sprint testing was conducted during the second session. At the start of the first session, the participant’s age, height, and body mass were recorded. Height was measured barefoot using a portable stadiometer (Seca, Hamburg, Germany), while body mass was recorded by electronic digital scales (Tanita Corporation, Tokyo, Japan). Each session featured the same dynamic warm-up, which consisted of a 5-min jog at a self-selected pace on a treadmill, 10 min of dynamic stretching, and progressive speed runs over 20 m. Following this, participants either progressed into the 5RM BSS strength or 20-m sprint testing. Participants wore their own athletic trainers for all tests, but no knee wraps, weightlifting belts, or other supportive garments were permitted during the BSS. Participants were assessed at the same time of day for both testing sessions, and refrained from intensive lower-body exercise in the day prior to testing. Participants were permitted to consume water as required throughout the sessions.

### 2.3. 5RM Bulgarian Split-Squat (BSS) Strength Testing

**Figure 1:**
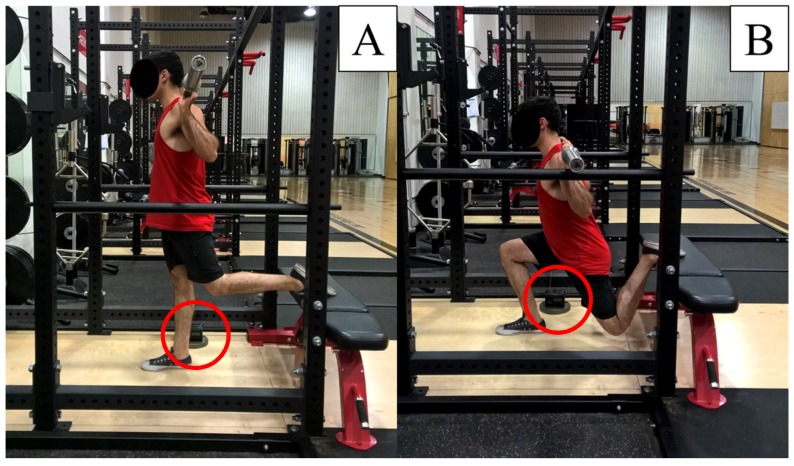
Start and finish (**A**) and bottom (**B**) positions in the Bulgarian split-squat when performed by the right leg. The linear position transducer is circled in (**A**) and (**B**).

The procedures for the BSS were adapted from McCurdy et al. [23], and performed with a standard Olympic bar and plates within a power rack (American Barbell, San Diego, CA, USA). As stated, the 5RM load was selected as it has been recommended and used within strength training [31,32,33], and has practical application for practitioners. Therefore, it was adopted in this study. A standard gym bench (American Barbell, San Diego, CA, USA) supported the leg not completing the lift, and was positioned so that participants could place the top of the foot on the bench to ensure the working leg was isolated to perform the BSS (Figure 1). Both legs were assessed, and the order of which leg was tested first was randomized amongst the sample. To determine the 5RM, participants completed 10 repetitions using approximately 40% of the perceived 1RM (i.e., the maximal load that the subject estimated that they could lift in the BSS) on the first set, followed by a set of five repetitions after adding 10–20% more weight. This is standard practice in strength testing research [3,4,34,35,36,37,38,39]. Following this, participants completed their first attempt at the 5RM [23]. This process continued until the participants were unable to successfully perform a 5RM, which typically occurred within five attempts.

Participants were instructed to descend until the top of the thigh of the working leg was parallel to the floor before ascending. This was visually assessed by the investigators, and participants were given verbal cues on when they were to halt the down phase, and begin the up phase, of the BSS [3,6]. The pins were adjusted in the rack and placed as close as possible to the bottom of the final position of the bar. The second leg was tested immediately after the first, and 3 min of recovery time was provided between collective attempts. Following the guidelines of McCurdy et al. [23], the investigators observed the participant’s working leg and the barbell for proper technique (i.e., the load was being driven up during the concentric phase by the working leg). If posterior displacement of the barbell occurred on the descent with no anterior movement of the knee joint (as this technique distributes more weight to the supported leg) [23], the lift was determined to be unsuccessful. The same load was lifted by both legs; failure on one leg resulted in test termination (i.e., the ‘weaker’ leg could be the limiting factor). However, even though the load may have been limited by the weaker leg, the stronger leg could have been more effective at generating force and power. This was a focus of the current study, and why these procedures were adopted. Failure to descend to the appropriate position for all repetitions also terminated the test. 

Power applied to the bar was measured during the BSS by a GymAware Powertool linear position transducer (Kinetic Performance Technology, Canberra, Australia). The transducer featured a spring-loaded retractable cable that passed around a spool integrated with an optical encoder [40]. The external end of the cable was attached on the inside of the barbell, and the transducer was then placed on the floor directly underneath the bar. The magnetic bottom was positioned on top of a weight plate to ensure the unit did not move. The cable provided no resistance to the bar, while the encoder recorded the movement of the bar for every 3 mm of bar movement. The linear position transducer recorded data at a frequency of 50 Hertz [22]. Data for each repetition was collected and stored on an iPad handheld device (Apple Inc., Cupertino, CA, USA), before being uploaded to an online database. Data was then extracted from this database. According to manufacturer guidelines [41], the raw displacement data has been deemed to be reliable and valid and is not treated within the software prior to being used in calculations for variables such as power and force. The mass of the barbell was entered into the software to allow calculation of the required variables, which included peak and mean power (force × velocity), velocity (bar displacement × time), and force (bar mass × acceleration). Data for the best repetition, and the average from each of the five repetitions, were recorded for peak and mean power, velocity, and force for both the left and right legs. Power, force, and velocity are variables commonly utilized by practitioners to provide practical information for coaches and athletes [19,21,22,40,42,43], and thus were used in this study. Although Lake et al. [44] suggested that barbell kinematics should not be used to derive power when measured by a motion capture system, Lockie et al. [21] reviewed literature regarding the GymAware Powertool and found relative consensus regarding reliability. For example, Black [41] reported typical errors of measurements for distance of 0.00 m, duration of 0.01–0.02 s, and velocity of 0.01 m·s^−1^. Hori and Andrews [45] reported high and acceptable reliability for peak velocity (coefficient of variation = 1.1–4.6%). Drinkwater et al. [19] detailed that concentric power had low coefficient of variations equaling 1.0–3.02% across a range of strength exercises. More recently, Banyard et al. [46] found that peak and mean velocity and force measured by a GymAware unit during the back squat were highly valid across loads from 20–100% 1RM when compared to data recorded from four linear position transducers and a force plate. Furthermore, Banyard et al. [46] found that mean and peak power were highly valid for all loads from 40–100% 1RM. As a result, all variables were considered reliable and valid for this study.

Only concentric variables were considered in this study, due to the importance of concentric force development for sprint acceleration [2]. For each mechanical variable analyzed in this study, the stronger or dominant leg was defined as that with the greater value; the weaker or non-dominant leg had the lower value [14,15]. This process was used as previous research has noted that leg dominance is task dependent [47,48,49], and thus should be defined specifically for each of the assessed variables. Between-leg differences used when determining the Spearman’s correlations in the mechanical variables were calculated as the percentage difference between the stronger and weaker legs (i.e., which leg recorded a higher value for power, force, or velocity) [14,15]. The formula [(*strong leg − weak leg)/strong leg] × 100* was used [14,15].

### 2.4. 20-m Sprint Testing

20-m sprint time was recorded by a timing lights system (Fusion Sports, Brisbane, Australia). Gates were positioned at 0 m, 5 m, 10 m, and 20 m, at a height of 1.2 m and width of 2.5 m, to measure the 0–5 m, 0–10 m, and 0–20 m intervals. Sprints over 5 m [6,9,50], 10 m [6,9,15,50], and 20 m [15,50] have been used in the assessment of running speed in men. Participants began the sprint from a standing start 50 cm behind the start line to trigger the first gate, started in their own time, and were instructed to maximally sprint through all timing gates. Time for each interval was recorded to the nearest 0.001 s. Two trials were completed [1,51], with the fastest trial used for analysis. The recorded times for the three intervals (0–5 m, 0–10 m, and 0–20 m) in the fastest trial were then used to calculate velocity through the equation *velocity = displacement·time^−1^* [9].

### 2.5. Statistical Analysis

Statistical analyses were processed using the Statistics Package for Social Sciences (Version 22.0; IBM Corporation, New York, NY, USA). Means ± standard deviations (SD) were calculated, in addition to 95% confidence intervals (CI). Stem-and-leaf plots were used to determine whether there were any outliers in the data for each variable [21,22,52,53], and outliers were treated via a winsorization method [21,22,53,54,55,56]. Paired samples t-tests were used to calculate any significant differences between the dominant and non-dominant legs for peak and mean power, force, and velocity for the BSS. Significance was set as *p* < 0.05. Effect sizes (*d*) were also calculated for the between-leg comparisons, where the difference between the means was divided by the pooled SD [57]. A *d* that ranged from 0.2 to 0.5 was considered a small effect; 0.5 to 0.8 a moderate effect; and 0.8 and above a large effect [58]. Due to the sample size [59], Spearman’s rank order correlation analysis was used to compute relationships between the dominant and non-dominant between-leg differences in the BSS mechanical variables (i.e., power, force, and velocity) and velocity in each 20-m sprint interval. Due to the high number of variables correlated, and to reduce the chances of making Type I errors, significance was set as *p* ≤ 0.01 [21,60]. The correlation coefficient strength was designated as per Hopkins [61]. 

## 3. Results

The mean BSS load lifted by the participants was 50.58 ± 16.69 kg. The mean velocity for the 0–5 m, 0–10 m, and 0–20 m intervals was 4.65 ± 0.22 m per second (m·s^−1^), 5.47 ± 0.20 m·s^−1^, and 6.39 ± 0.20 m·s^−1^, respectively. Table 1 displays the power, force, and velocity data for the dominant and non-dominant legs as defined by each BSS mechanical variable, as well as the between-leg percentage differences for all variables. The paired samples t-test analysis indicated that the dominant leg was significantly superior in 11 of the 12 mechanical variables, with only the set average for mean force not reaching significance. The effects for the power variables ranged from small-to-moderate; for the force variables, trivial-to-small; and for the velocity variables, moderate-to-large. 

The correlation data is shown in Table 2. There was a negative, large relationship between the between-leg difference in the best repetition for mean force and 0–5 m velocity. This result indicated that a higher velocity was associated with a lower difference between the dominant and non-dominant legs in mean force. Nevertheless, this correlation did not reach significance (*p* = 0.015), and no other BSS variable correlated with velocity over any of the sprint intervals.

## 4. Discussion

This study provided a preliminary investigation of the use of the BSS to identify between-leg differences in mechanical variables such as peak and mean power, force, and velocity in strength-trained men. Additionally, the relationship between these variables and 20-m sprint performance was calculated. Although the current research was exploratory in nature, there is currently no research that has investigated the mechanics of the BSS with a view towards measuring between-leg asymmetries in strength. Further to this, there is limited research that has investigated the influence that between-leg strength or power asymmetries could have on sprint acceleration [12,15,62], so this study adds to the body of knowledge in that area. The results from this study indicated that the BSS can be used to document between-leg differences in lift mechanics when comparing the dominant and non-dominant legs. Although there were significant differences between the legs for power, force, and velocity, the subjects in this study were still generally below the functionally significant difference asymmetry level of 15% [17,18]. Furthermore, there was only one significant relationship out of 36 correlations for the between-leg differences in BSS mechanics and velocity over the 0–5 m, 0–10 m, and 0–20 m sprint intervals. Despite this, there are still practical applications for the strength and conditioning coach and sport scientist that can be drawn from this introductory research.

It is important to identify between-leg strength differences, especially in athletic populations. If strength asymmetries do exist in an individual, this could negatively impact short sprint performance, whether the individual is accelerating from a stationary position or out of a change-of-direction or cut [13]. In addition to this, the individual may place too much emphasis on the stronger limb and increase their risk of injury [63]. Previous research has indicated leg dominance is task-specific [47,48,49], so the stronger leg in each variable was defined as the leg with the highest power, force, or velocity applied to the bar. The results from this study showed that for the mechanical variables of peak and mean power, force, and velocity, in both the best repetition and mean across the set, the dominant leg performed significantly better. Therefore, even though the same load was lifted, the better performing leg tended to have a higher bar velocity, and the greater resulting power. These results highlight the importance of the quality of the lift as opposed to just the load lifted, as higher bar speeds could lead to better power adaptations during resistance training programs.

However, even with the significant differences between the dominant and non-dominant legs, the asymmetries only ranged from approximately 0.3–12% in strength-trained men. As noted, all of these variables were below 15%, which is said to be the limit where an asymmetry becomes an issue [17,18]. Indeed, the greatest difference (set average for mean power = 12.35 ± 10.12 watts) was still below this value. It is important to note that although individuals may strive for balance between the limbs, strength imbalances between the legs can still exist in trained athletes [14]. Future research could also investigate the actual difference in maximal load lifted by the dominant and non-dominant legs in the BSS, and this method could also be used for those practitioners that do not have access to linear position transducers. Nevertheless, for practitioners that have access to this type of equipment, a linear position transducer in the BSS can measure mechanical variables such as power, force, and velocity that can provide a profile of between-leg strength differences in trained individuals. Given the practical nature of measuring the mechanics of a strength exercise with a linear position transducer [19,20,21,22], this is beneficial information for the strength and conditioning coach.

The fact that there were relatively small between-leg differences in the BSS mechanics from the sample in this study would have likely contributed to the lack of significant relationships with 20-m sprint performance (Table 2). This was counter to the study’s hypothesis. However, the results from this study provide support to Lockie et al. [15], who suggested that if any between-leg strength differences are relatively low (i.e., below 15%), then this should not impact linear sprinting speed. This appears to be the case in the current research, and is useful information for the strength and conditioning coach. Generally in resistance training, the coach attempts to develop strength symmetry between the legs in their athletes. If this is successfully attained, this would be more likely to positively contribute to acceleration, as long as the athlete has the requisite technique [32] and neuromuscular coordination and control [39] required for short sprint performance.

There was one large correlation, which although non-significant, was between the best repetition for 5RM BSS mean force between-leg difference and 0–5 m velocity. The relationship suggested that a higher velocity was associated with a lower difference between the dominant and non-dominant legs in mean force. This large relationship provided some support to previous research that demonstrated that smaller differences in eccentric knee flexor torque related to faster 40-m sprint performance in male team sport athletes [15]. Indeed, one of the key issues for an athlete in transferring force to the sprint step is ensuring that the nervous system can control the augmented strength to recruit the appropriate motor units for the sprinting activity [39]. Nevertheless, this correlation did not reach significance, nor did any other relationship between sprint velocity and BSS between-leg mechanics. The results from this study do not discredit the value of measuring the between-leg mechanics of the dominant and non-dominant leg in the BSS, with a view to investigating the impact of any asymmetries on athletic performance. Rather, they highlight the fact that relative symmetry between the legs can limit any negative impacts on a physical capacity such as sprint acceleration performance in trained men.

There are several study limitations that should be acknowledged. The sample size was small (*n* = 8), which meant that this research could only provide an initial exploratory analysis of the relationships between the mechanics of the BSS and sprint acceleration. This also meant that the majority of participants were below the clinically significant between-leg strength difference of 15% [17,18]. Indeed, depending on the mechanical variable, there was only a maximum of 1–2 subjects that had any differences greater than 15%. Future research should use a larger sample to see if individuals who have between-leg differences in peak and mean power, force, and velocity as measured by the BSS above 15% experience negative impacts on sprint acceleration performance. This could also incorporate the analysis of muscle or fat-free mass in the lower limbs, and whether this influences power, force, and velocity. Although the participants in this study were strength-trained men, they were not high-level field or court sport athletes. Participants with this background may exhibit different relationships between the BSS and 20-m sprint. A linear position transducer was used to measure data in this study due to its wide use and practical application in the field [19,20,21,22]; however, a force plate can directly measure the kinetics of the BSS. This should be explored in future studies. Only one unilateral strength exercise was measured in this study. Other exercises, such as the step-up or lunge, may provide different results. Furthermore, isokinetic strength testing has been used to identify between-leg strength differences that can affect running speed [15]. Future research could compare whether unilateral isokinetic strength tests are comparable to an isotonic exercise such as the BSS. However, the BSS was chosen because of its use in training [23,25,26], and since it can be easily measured with a linear position transducer. This provides practical application for the results of this study.

## 5. Conclusions

In conclusion, and within the context of the study limitations, the BSS can produce power, force, and velocity data that profile between-leg differences for the dominant and non-dominant legs in strength-trained men. Furthermore, individuals who are strength trained appear to be well positioned to maintain any between-leg strength differences below 15%, which should limit any negative impacts to athletic activities such as sprinting. Indeed, this study showed limited relationships between the between-leg differences in peak and mean power, force, and velocity in the BSS, and 0–5 m, 0–10 m, and 0–20 m sprint velocity. This was likely influenced by the fact that the sample demonstrated relative symmetry between the legs in the BSS mechanical variables. Future research should examine a larger sample with participants who display between-leg differences in BSS peak and mean power, force, and velocity in excess of 15% to determine whether this has a negative impact on sprint performance.

## Figures and Tables

**Table 1:** Descriptive data (mean ± SD; 95% CI) for best repetition and set average, and between-leg comparisons, for dominant and non-dominant leg peak (PP) and mean (MP) power, peak (PF) and mean (MF) force, and peak (PV) and mean (MV) velocity, for the 5RM Bulgarian split-squat in strength-trained men (*n* = 8). Power was measured in watts; force was measured in newtons; velocity was measured in meters per second.

Variable	Dominant Leg	Non-Dominant Leg	*p*	*d*	Difference
PP Best Repetition	999.79 ± 166.36	913.76 ± 150.66 *	0.014	0.54	8.39 ± 7.48
	(860.71–1138.87)	(787.80–1039.72)			(2.13–14.65)
PP Set Average	922.54 ± 144.89	838.61 ± 123.55 *	0.005	0.62	8.82 ± 6.52
	(801.41–1043.68)	(735.32–941.89)			(3.37–14.27)
MP Best Repetition	678.75 ± 104.69	603.39 ± 86.52 *	0.01	0.78	10.75 ± 8.02
	(591.23–766.28)	(531.05–675.72)			(4.04–17.45)
MP Set Average	630.03 ± 96.94	603.38 ± 86.52 *	0.01	0.29	12.35 ± 10.12
	(548.98–711.08)	(487.26–608.39)			(3.88–20.80)
PF Best Repetition	1754.48 ± 273.43	1675.22 ± 218.15 *	0.032	0.32	4.20 ± 3.56
	(1525.89–1983.07)	(1492.84–1857.61)			(1.23–7.18)
PF Set Average	1684.87 ± 260.08	1608.36 ± 236.31 *	0.012	0.31	4.45 ± 3.27
	(1467.44–1902.31)	(1410.80–1805.92)			(1.72–7.18)
MF Best Repetition	1274.29 ± 201.54	1266.59 ± 196.70 *	0.013	0.04	0.57 ± 0.40
	(1105.80–1442.79)	(1102.14–1431.03)			(0.24–0.91)
MF Set Average	1264.94 ± 200.15	1261.17 ± 196.79	0.056	0.02	0.28 ± 0.29
	(1097.62–1432.27)	(1097.62–1432.27)			(0.03–0.52)
PV Best Repetition	0.74 ± 0.07	0.68 ± 0.09 *	0.002	0.74	7.79 ± 5.25
	(0.68–0.80)	(0.61–0.75)			(3.40–12.19)
PV Set Average	0.69 ± 0.08	0.63 ± 0.08 *	0.003	0.75	8.97 ± 5.98
	(0.62–0.76)	(0.56–0.69)			(3.97–13.96)
MV Best Repetition	0.54 ± 0.05	0.49 ± 0.08 *	0.006	0.75	10.27 ± 8.11
	(0.50–0.58)	(0.42–0.55)			(3.49–17.05)
MV Set Average	0.50 ± 0.06	0.44 ± 0.07 *	0.006	0.92	12.57 ± 9.83
	(0.46–0.55)	(0.38–0.50)			(4.35–20.78)

* Significantly (*p* < 0.05) lower than the dominant leg.

**Table 2:** Spearman’s correlations (*ρ*) between the between-leg percentage differences between the dominant and non-dominant legs in best repetition and set average for peak (PP) and mean (MP) power, peak (PF) and mean (MF) force, and peak (PV) and mean (MV) velocity from the 5RM Bulgarian split-squat, with velocity from the 0–5 m, 0–10 m, and 0–20 m intervals in strength-trained men (*n* = 8).

Variable		0–5 m	0–10 m	0–20 m
PP Best Repetition	*ρ*	0.19	0.167	0.143
	*p*	0.651	0.639	0.76
PP Set Average	*ρ*	0.143	0.143	0.286
	*p*	0.736	0.736	0.535
MP Best Repetition	*ρ*	−0.048	−0.048	0.036
	*p*	0.911	0.911	0.939
MP Set Average	*ρ*	0.012	0.048	0.234
	*p*	0.978	0.91	0.613
PF Best Repetition	*ρ*	0.119	0.405	0.393
	*p*	0.779	0.32	0.383
PF Set Average	*ρ*	0.143	0.381	0.464
	*p*	0.736	0.352	0.294
MF Best Repetition	*ρ*	−0.810	−0.476	−0.071
	*p*	0.015	0.233	0.879
MF Set Average	*ρ*	−0.407	0.06	0.378
	*p*	0.317	0.888	0.403
PV Best Repetition	*ρ*	0	−0.048	0
	*p*	1	0.911	1
PV Set Average	*ρ*	0.143	0.143	0.286
	*p*	0.736	0.736	0.535
MV Best Repetition	*ρ*	−0.071	0.119	0.214
	*p*	0.867	0.779	0.645
MV Set Average	*ρ*	0.12	0.192	0.396
	*p*	0.778	0.649	0.379

## Abbreviations

m	Meters
BSS	Bulgarian split-squat
5RM	Five repetition-maximum
kg	Kilograms
SD	Standard deviation
CI	Confidence intervals
*p*	Significance
*d*	Effect size
*ρ*	Spearman’s rho correlation coefficient
m·s^−1^	Meters per second
PP	Peak power
MP	Mean power
PF	Peak force
MF	Mean force
PV	Peak velocity
MV	Mean velocity
